# Agent-Based Medical Health Monitoring System

**DOI:** 10.3390/s22082820

**Published:** 2022-04-07

**Authors:** Mamoona Humayun, Noor Z. Jhanjhi, Abdullah Almotilag, Maram Fahhad Almufareh

**Affiliations:** 1 Department of Information Systems, College of Computer and Information Sciences, Jouf University, Sakaka 42421, Saudi Arabia; abdull@ju.edu.sa (A.A.); mfalmufareh@ju.edu.sa (M.F.A.); 2 School of Computer Science and Engineering (SCE), Taylor’s University, Subang Jaya 47500, Malaysia; noorzaman.jhanjhi@taylors.edu.my

**Keywords:** healthcare, agent, chronic disease, health monitoring, e-health solution

## Abstract

One of the leading healthcare concerns worldwide is the aging population. Aged patients require more significant healthcare resources because they are more likely to have chronic diseases that result in higher healthcare expenses. The design and implementation of e-health solutions, which offer patients mobile services to assist and enhance their treatment based on monitoring specific physiological data, is one of the key achievements in medical information technology. In the last few decades, there have been tremendous advancements in healthcare technology regarding mobility, size, speed, and communication. However, the critical drawback of today’s e-Health monitoring systems is that patients are confined to smart rooms and beds with monitoring gadgets. Such tracking is not widespread due to chronic patients’ mobility, privacy, and flexibility issues. Further, health monitoring devices that are fastened to a patient’s body do not give any analysis or advice. To improve the health monitoring process, a multi-agent-based system for health monitoring is provided in this study, which entails a group of intelligent agents that gather patient data, reason together, and propose actions to patients and medical professionals in a mobile context. A multi-agent-based framework presented in this study is evaluated through a case study. The results show that the proposed system provides an efficient health monitoring system for chronic, aged, and remote patients. Further, the proposed approach outperforms the existing mHealth system, allowing for timely health facilities for remote patients using 5G technology.

## 1. Introduction

The rapidly increasing aging population is a significant challenge in the current times. According to the recent demographic trends, the number of elderly individuals will continue to increase dramatically [1]. According to [2], the number of individuals aged 65 and more is expected to reach about 1.5 billion by 2050, with most growth occurring in emerging nations. These demographic shifts necessitate a shift in worldwide focus to address the senior population’s preventive healthcare and medical demands. E-health offers the great promise of providing health facilities to aged and chronic people by enhancing the reach of healthcare facilities to remote patients [3,4]. E-health systems based on the latest cutting-edge technologies are beneficial. Still, the challenge lies in integrating this system with the healthcare needs of chronic and aged patients in a cooperative way [3,5]. This necessitates the development of a computational framework that allows collaborative work. One of the suitable ways to integrate technology with health monitoring activities is to create artifacts that can cooperate in providing timely healthcare to remote patients [6]. To provide a unified and efficient solution to this problem, we have developed a multi-agent-based framework in this study for providing timely healthcare services to remote patients. The agent in our framework is a software entity that works as a cooperative artifact and facilitates the patients and hospital staff.

**Figure 1:**
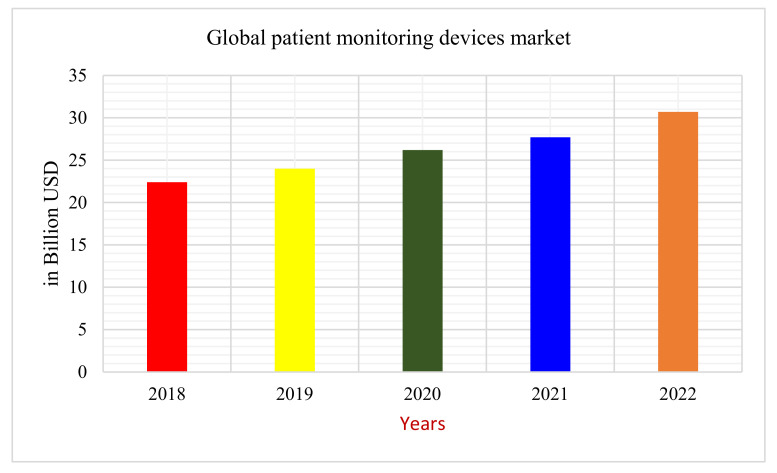
Global market of patient monitoring devices [13].

A multi-agent system is a group of autonomous agents that interact to coordinate their activities. They may collectively solve a problem that no one agent could solve alone [7]. The proposed framework uses a multi-agent-based system. These different agents communicate and provide timely health services to the patients remotely. The development of wearable and implanted sensors has significantly improved healthcare over several decades [8,9,10]. The global market for patient monitoring devices is also growing fast, as shown in Figure 1. These devices have made remote patient monitoring simple by allowing for real-time monitoring. However, the disadvantage of these devices is that they just gather the patient’s vital signs and cannot take any action in an emergency [10,11,12]. The remote patient agent in the proposed study will overcome this limitation by sending the patient’s vital signs to the Doctor agent.

This research contributes to patient health monitoring in multiple ways. Firstly, it will provide a cooperative working environment where various agents will work together to provide timely health services to remote patients. Secondly, the provided system will help leverage the potential benefits of wearable and implantable devices by timely collecting and monitoring vital signs and sending them to the healthcare professional for suitable action. Third, the agent will share the workload of hospital staff by sending only the abnormal vital signs to the caretaker. Lastly, the proposed solution will not only serve the chronic and aged patients in the urban area, but it will also facilitate the patients living in rural areas where there is a scarcity of specialized medical staff and modern healthcare facilities.

The remainder of the paper is organized as follows: Section 2 will provide an overview of the existing medical health monitoring system (HMS). Section 3 will provide a multi-agent-based framework for monitoring patients and providing timely health care services. Section 4 will evaluate the proposed framework with the help of a case study. Section 5 will discuss the findings of the current study along with its comparison to the existing study. Section 6 will provide the conclusion of the current research study by providing insights into future work. Table 1 lists abbreviations used in this study.

## 2. Literature Review

This section will review previous research on healthcare monitoring systems to determine the present state of the art and any shortcomings.

A multi-agent-based mobile HMS is proposed in [14], which combines data mining techniques with a wireless medical sensor module. The proposed scheme facilitates communication between patients, physicians, and other healthcare employees. This study uses two types of data: real-time sensory data from the patient’s body and historical data obtained in the past. This system gathers diagnosis patterns, categorizes them into normal and emergency categories, and then declares an emergency by comparing the two data groups previously described. As a result, approaches for analyzing and modeling patterns of normal and emergency patient states are proposed.

Paper [3] proposed an awareness model to show how software agents can play the roles of humans in mobile health monitoring. It discusses the challenges involved in mobile HMS and proposes a layered framework based on these identified challenges. The framework helps understand the role of agents in HMS; however, no validation was performed to ensure the authenticity of the proposed framework.

The architecture of an HMS based on WSN, capable of collecting, retrieving, storing, and analyzing the patient’s vital signs, is presented in [15]. Proposed MAS is utilized to handle these sensors and gather and store data in a database. The agents’ dynamic nature and mobility make them ideal for keeping these sensors alive in the WSN. There are four agents in the planned MAS named AA, CA, QA, and DA. The AA is responsible for invoking and terminating other agents. The DA handles data reduction. The QA provides the vital signs information. The suggested MAS has been implemented in the JADE environment using Java, and the results have been verified.

Paper [16] has presented an NMAA for monitoring local and remote patients using mobile technology. The Paper highlights the importance of using software agents in providing better healthcare. The proposed framework also facilitates information exchange between the members of health staff and patients and provides a solution to handle the problems of emergency and chronic patients. The proposed approach was beneficial at that time, but with the advancement in technology and infrastructure, there is a need to develop a more secure and efficient architecture based on existing CET.

Paper [17] proposes an intelligent integrated system (IIS) to store and retrieve medical records on clouds. The proposed IIS collects data from medical sensors, converts it into s standard format, and stores it on a cloud internet. The patients can initiate/send audio/visual data to the medical personnel through this system. An SLR was performed in [18] to explore the role of agents in the psychiatry field for careening, diagnostics, and treatment of psychological problems. The results of SLR show that the use of agents is favorable in aiding patients with mental illnesses. A novel method of power optimization was proposed in [19], which uses SOM and multi-agent-based DAI to improve the efficiency of WSN, which is widely used for interconnecting multiple health sensors. Network lifetime is a problem in WSN, due to the limited power of the monitoring sensor; the proposed approach results in better performance of WSN as compared to existing approaches. The results of the study were validated using mathematical analysis and simulation.

Another article [20] created a MAS for bridging the gap between patient and physician. The proposed system uses intelligent agents’ services for serving patients at home. The proposed system was validated by creating a prototype. Results show that the developed agents can select a suitable plan and communicate with a physician for validation and permission. An agent-based framework for teamwork in healthcare is presented in [21]; the proposed framework creates a team of interdisciplinary healthcare professionals for treating chronic or emergency patients. However, the problem with this approach is that the team’s structure varies depending on the patient’s complexity. Despite this, the proposed research is helpful in better understanding the role of teamwork in healthcare and the way to design it.

Paper [22] examined the management of medical data flows in IoT-based healthcare networks. A priority-based queueing system is created and evaluated to describe the dynamic nature of wireless transmission scheduling while adhering to the medical-grade quality of service (QoS) guidelines. Considering the intelligence of gateways in IoT-based beyond-WBAN, a truthful and efficient mechanism is proposed, TMDC, that can ensure that all gateways accurately report the actual priority levels of their medical packets while incentivizing the base station to participate in beyond-WBAN scheduling. According to theoretical and simulation studies, the proposed mechanism may satisfy all design requirements and outperform its competitors in packet transmission probability and network profitability.

The preceding discussion and the comparison provided in Table 2 show that agents can play a vital role in patient health monitoring by providing various services such as facilitating communication between patients and physicians, patient counseling, transmitting vital signs to physicians, etc. However, there is a need to develop a comprehensive MAS framework to help indoor and outdoor patients. It may also facilitate the patients living in remote areas with few healthcare facilities. To fill this gap, this research work has proposed a framework ABHCMF for monitoring patients in the hospital, patients with wearable devices at home, and patients living in remote areas with fewer healthcare facilities. The details of ABHCMF are given in the section of the proposed methodology.

## 3. Proposed Methodology

**Figure 2:**
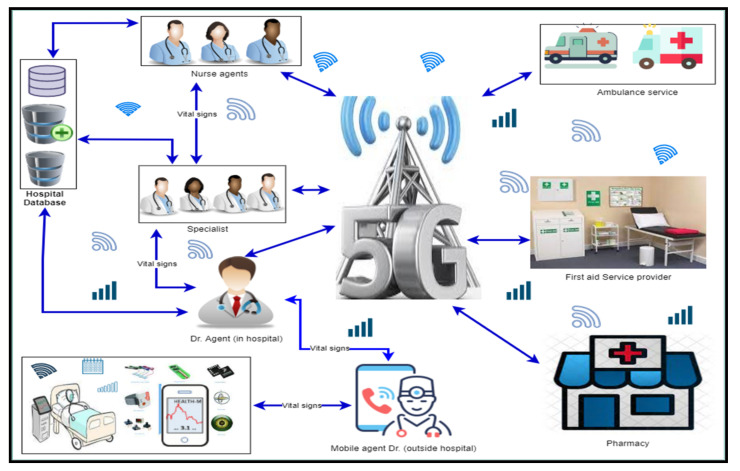
Proposed Framework ABHCMF.

This section involves proposing a framework for healthcare monitoring of aged and chronic patients based on the analysis of existing data. The proposed framework ABHCMF aims to improve patient health monitoring using a multi-agent system. The key features of the proposed framework over existing approaches are many; first, reducing the workload of doctors in the hospital by transferring only the abnormal vital signs to the doctor through the mobile agent. Second, the specialist workload will also be reduced as the doctor will only refer the patient to the concerned specialist in case of critical issues. Third, the doctor can provide timely healthcare service to the patient by instantly sending messages to the first aid service or ambulance through 5G. Lastly, the proposed system will facilitate the patients living in rural areas with fewer healthcare facilities by providing remote recommendations through 5G. A detailed overview of the framework is depicted in Figure 2. Below we discuss the essential use cases of ABHCMF.

### 3.1. ABHCMF Use Cases

The proposed ABHCMF framework consists of four main agents, namely, Mobile Agent Doctor (outside the hospital) named (MADO), Doctor Agent inside the hospital (DAH), Nurse Agent (NA), and Pharmacy Agent (PA). Further, there are two core modules named Patient vital signs and Specialist doctor. In the following subsection, we describe the working of each agent and module.

#### 3.1.1. Patient Vital Sign

**Figure 3:**
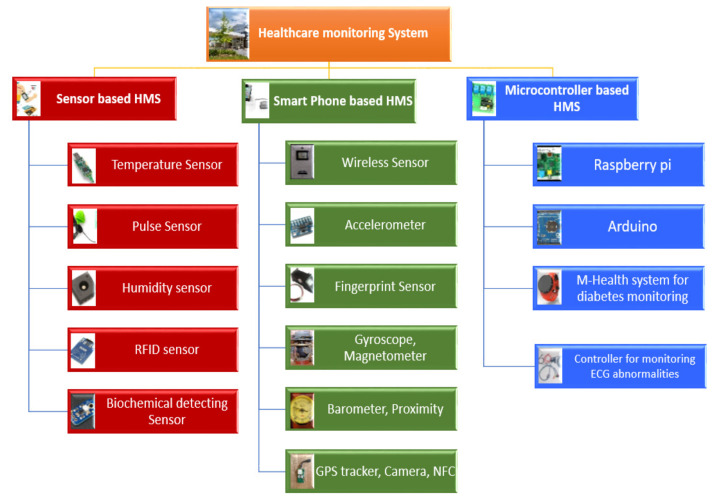
A taxonomy of sensors used for healthcare monitoring.

Our framework’s fundamental module is the patient, as the entire framework was built to make the patient’s life easier. The patient is an elderly or chronic patient located at a remote place and has wearable sensors attached to their body. These wearable sensors are connected to MADO over the internet and retrieve the patient’s vital signs through a smart device. The patient’s smart device is not intelligent, so it simply fetches the patient data and transmits it to the MADO for further processing. Figure 3 provides the taxonomy of health monitoring sensors to better understand the various types of health monitoring sensors.

#### 3.1.2. Mobile Agent Doctor Outside Hospital (MADO)

MADO obtains patient vital signs via the patient’s smart device, which takes vital signs from several wearable sensors attached to the patient’s body. It checks the vital signs’ values and sends them to the DAH only if the signs vary from the normal data values stored in MADO memory. However, MADO does not have this much intelligence to decide or suggest some recommendations for patients. It simply transmits the vital signs to the DAH through 5G or Wi-Fi.

#### 3.1.3. Doctor Agent in Hospital (DAH)

**Figure 4:**
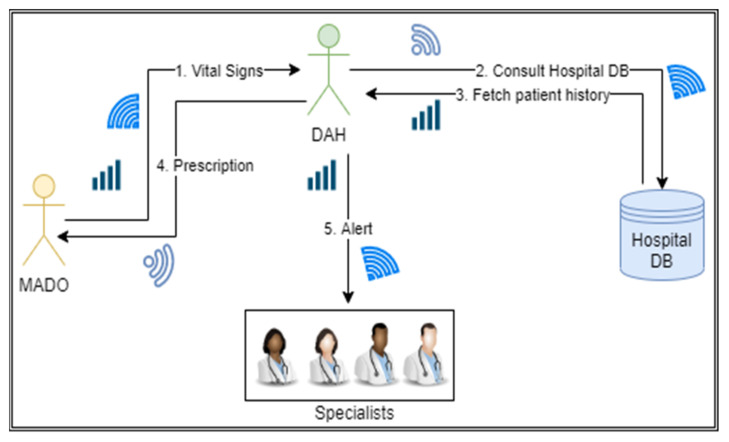
Role of DAH in providing healthcare facilities.

It is one of the core agents in the proposed ABHCMF, which receives a patient’s vital signs from MADO through the internet and decides accordingly. DAH serves as an intermediary between MADO and specialists. DAH agent reduces the specialist agent’s workload by sending only the abnormal cases to the specialist doctor. This DAH agent is connected to MADO, specialist agent, hospital database, and emergency services through 5G. DAH receives patient’s vital signs through a mobile agent and analyses these vital signs. It also checks the patient’s history from the hospital database; if the vital signs are a little bit above or below the desired range, it sends a prescription to the MADO, which is further transmitted to the patient on the smart device. Otherwise, DAH chooses the concerned specialist and communicates the vital signs to the specialist for further action. The working of DAH is shown in Figure 4.

#### 3.1.4. Pharmacy Agent (PA)

PA is the side/sleeping agent of the proposed model, which invokes only if it receives some prescription through SA and provides prescribed medicines to the patient or the one who brings the prescription. PA is also connected to the hospital’s online application, from where it can check the medications prescribed by the doctor for a particular patient. PA is only allowed to provide the prescribed medicines to the patient.

#### 3.1.5. Nurse Agent (NA)

This agent is exclusively responsible for indoor patients sent to the hospital by SA for immediate treatment. NA has access to the hospital database from where it can check the history of the patients. It acts following DAH and SA’s directions.

#### 3.1.6. Specialist

**Figure 5:**
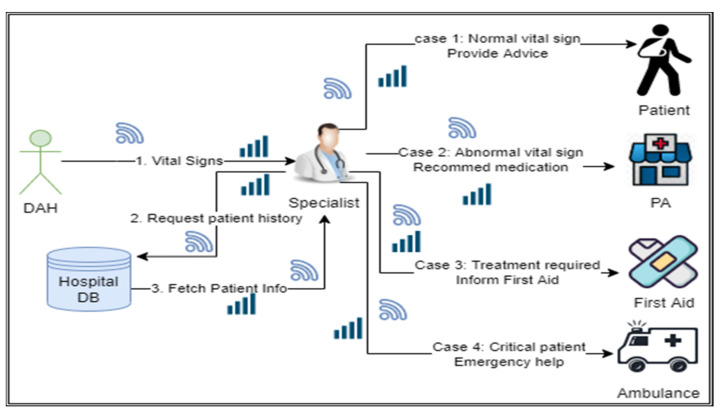
Role of the specialist in providing healthcare facilities.

The specialist is the health professional expert in a particular area/specialization [23]. This agent is connected to MADO, DAH, hospital database, PA, and emergency services through the 5G network. The specialist receives vital signs for the patient from DAH and responds appropriately; it also has access to the hospital database, which it uses to look up patient information. If the received vital signs are normal and there is no need for medication or treatment, SA sends advice directly to the patient. If the received vital signs are not normal, SA prescribes the medication and forwards it to PA, which then sends intimation to the patient on smart device. If the vital signs indicate that patient needs immediate care/treatment, SA sends the alert to nearby first aid for patient assistance. In a critical situation, SA sends alerts to the emergency help service (ambulance) to bring patients to the hospital and send alerts to nearby hospitals. The detailed working of SA is shown in Figure 5.

## 4. Evaluation of Proposed Methodology Using Case Study of Existing Patients

This section will test the proposed framework with the help of a case study. Table 3 shows the data of some aged patients, including the vital signs of the patients and the medical history already stored in the database. As the proposed model was mainly designed for geriatric and chronic patients, agents will therefore compare the given vital signs with the normal range [24] of older people.

According to the data mentioned in Table 3, when the MADO compares Alice’s vital signs with the normal ranges, it finds that the data of a few vital signs is not normal, so it immediately transmits Alice’s data to the DAH. The DAH receives Alice’s vital signs and checks his history from the hospital database. When the DAH comes to know that Alice is a chronic patient having asthma and three out of five vital signs are abnormal, it simply refers him to a specialist. The specialist in our case is the human medical specialist. The specialist analyses the vital signs and checks the patient history from the database. Based on the analysis of vital signs and patient history, specialists choose one of the four options given in the proposed model. Specialists will either propose a piece of advice to the patient if he considers these vital signs to be normal or suggest some medicines in case of abnormal vital signs. Suppose the specialist feels that Alice needs an immediate medical checkup. In that case, he will communicate this to Alice on his smart device and intimate the nearby first-aid provider. If the specialist feels that Alice’s situation is critical, he will intimate the ambulance service nearby to bring him to the hospital.

Bob is a hypertension patient, so when the MADO realizes that the received vital signs of Bob do not fall in the normal range, it immediately transfers Bob’s data to the DAH. The DAH checks the history of Bob from the hospital database and comes to know that he is a hypertension patient. When the DAH observes the vital signs, it senses that the patient has a fever, short breathing, and high blood pressure. The DAH refers Bob to a cardiac specialist. The selection of specialists is based on the data of vital signs stored in the DAH’s memory and patient’s history. The specialist analyses the vital signs and patient history, determines the patient’s severity and takes one out of four actions mentioned in the proposed model. In the case of patient Jack, who has a normal medical history, when the MADO determines a little abnormality in his temperature, it transfers vital signs to the DAH. The DAH checks the patient history and knows that Jack has a normal medical record. The DAH observes the remaining vital signs, which are quite normal. In this case, the DAH can send some recommendations directly to the patient without consulting the specialist.

Mary is a diabetic patient wearing CGM (continuous glucose monitor) to monitor her glucose level; the MADO notes that the received vital signs are highly abnormal. It immediately sends the vital parameters to the DAH. The DAH checks the history of Mary and comes to know that she has diabetes; further, the pulse rate and body temperature are also high, along with blood sugar. The DAH transfer her data to the endocrinologist. The endocrinologist observes the vital signs of Mary along with her past diabetic history and makes a suitable decision. In the above case study, we may note that the MADO works as an intermediary bridge between the patient and healthcare staff. Further, the proposed methodology reduces the burden of healthcare staff by sharing responsibilities through agents.

## 5. Discussion

This research proposed a framework to facilitate medical healthcare monitoring by providing real-time tracking of aged, chronic and remote patients using a multi-agent system. The proposed framework was evaluated with the help of a case study by using the existing data of four patients. Three out of four patients were suffering from chronic diseases; below, we discuss how the agent system works by comparing the patient vital signs data with benchmark values set for the elderly persons. We take one patient with normal patient history and one chronic patient to discuss the working of our proposed framework.

**Figure 6:**
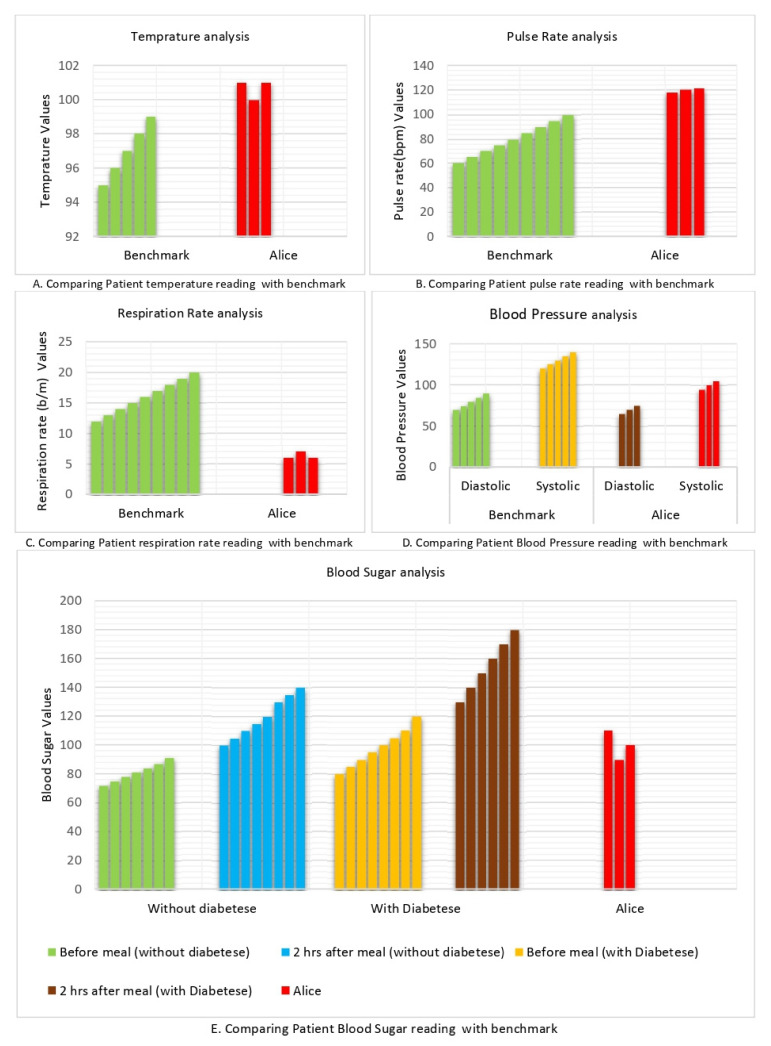
Handling chronic aged patient using proposed multi-agent HMS.

Alice is an asthmatic patient, the MADO obtains the reading of Alice’s vital signs three times a day, and MADO observes that a few of Alice’s vital signs do not fall in the normal range; for example, Alice’s temperature is above normal (see Figure 6A), her reading for pulse rate is also above the normal range (see Figure 6B), and her respiration rate is below normal (see Figure 6C), while other vital signs fall in the normal range, as can be seen from Figure 6C–E.

The MADO transmits this data to the DAH, the DAH has access to the hospital database. It checks Alice’s history and comes to know that he is an asthmatic patient. After observing multiple abnormal vital signs and patient history, the DAH transmits vital sign data of Alice to the specialist. The specialist received the vital signs of Alice and checked his past history from the hospital database. Based on the vital signs, patient history, and specialist’s knowledge, he takes one of the four actions mentioned in the proposed framework. If the specialist feels that Alice has the same vital signs in the past and it is pretty normal for him, he suggests some advice and sends it to Alice. If the specialist feels that Alice needs some medication, he prescribes the medicines and sends them to the PA and patient. If the specialist thinks that Alice needs a medical checkup, he informs the nearby first aid to assist Alice. If the specialist feels that Alice’s situation is critical and should be brought to the hospital, he will inform the ambulance service to carry Alice to the hospital. Thus, the proposed solution will provide timely help to Alice and save him from fatality.

**Figure 7:**
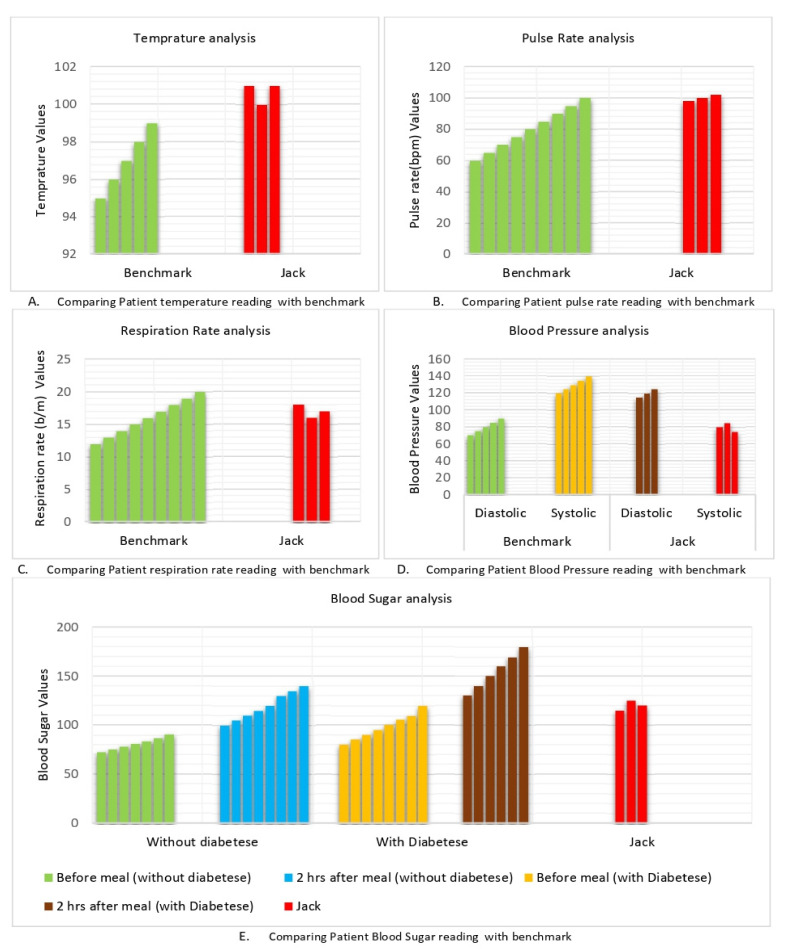
Handling normal aged patients using multi agent HMS.

Jack is an elderly patient with normal medical history. The MADO receives an abnormal temperature value for Jack, as shown in Figure 7A, while the remaining vital signs for Jack are normal (as shown in Figure 7B–E) the MADO transfers this data to the DAH. The DAH checks Jack’s history from the hospital database and comes to know that he has normal medical history. Further, the DAH analyses the vital signs and observes that only temperature is above normal while all other vital signs fall in the normal range. In such a case, the DAH prescribes medication/suggestions to Jack without involving a specialist. This shows that the proposed system shares the workload of healthcare staff.

## 6. Conclusions and Future Work

Healthcare technology has advanced dramatically in mobility, size, speed, and communication in the past few decades. However, the real-time monitoring and timely availability of health facilities to the aged and chronic patients is a challenge. Geriatric patients require more healthcare resources because they are more prone to develop chronic conditions, which lead to increased healthcare costs. E-health monitoring aims to provide real-time monitoring of these patients with the help of wearable devices. However, patients are restricted to smart rooms and beds with monitoring equipment, which is a major downside of today’s e-health monitoring systems. Further, smart devices/gadgets linked to the patient’s body that monitor their health give no analysis or advice. This research proposes a multi-agent-based framework for health monitoring, which comprises a collection of intelligent agents that acquire patient data, reason together, and suggest actions to patients and medical professionals in a mobile setting enhance the health monitoring process. The proposed system outperforms the existing system in many ways: First, it reduces the workload of doctors and specialists by introducing the concept of the mobile agent. Second, it connects all healthcare professionals using 5G technology. Third, it provides timely healthcare facilities to in-house patients and remote patients. Lastly, the proposed system offers emergency healthcare services by sending alarms to the emergency service providers (ambulance, first aid, and pharmacy). A case study is used to examine a multi-agent-based framework described in this research. The findings suggest that the proposed system can effectively monitor the health of chronic, elderly, and distant patients. In the future, we will apply the proposed framework in real health settings to validate its strength.

## Figures and Tables

**Table 1:** List of abbreviations used.

Abbreviations	Used for	Abbreviations	Used for
HMS	Health monitoring system	NMAA	Networked multi-agent architecture
WSN	Wireless sensor networks	CET	Cutting-edge technologies
MAS	Multi Agent-based System	IIS	Intelligent integrated system
AA	Admin agent	SLR	Systematic literature review
DA	Data agent	DAI	Distributed artificial intelligence
CA	Control agent,	SOM	Self-organizing maps
MADO	Mobile agent doctor outside the hospital	ABHCMF	Agent-based healthcare monitoring framework
QA	Query agent	DAH	Doctor agent inside the hospital
PA	Pharmacy agent	SA	Specialist agent
NA	Nurse agent	E-health	Electronic health

**Table 2:** Comparison of existing literature on agent-based HMS.

Paper#	Target Population	Technology Used	Proposed Solution	Pros	Cons	Research Gap
[14]	Local patients	Wireless medical sensor module with data mining techniques	Multi-agent based mobile HMS	Real-time monitoring of patients	The proposed agent just plays the role of data transmission	Biosensors security
[3]	Healthcare	Mobile health monitoring	Propose a Computer Supported Cooperative Work-based technique	Awareness about human roles in mobile health Monitoring activities using software agents.	The proposed agent just plays the role of data transmission	Validation was missing
[15]	Elderly people in indoor environments	Software agents	Agent-based health monitoring	Data reduction and energy saving	Only handle indoor patients	The main focus is on data reduction instead of patients care
[16]	Aged and chronic patients	Wireless mobile technologies	Agent-based health monitoring	Proactive healthcare Enhanced patient-doctor interaction Enhanced information exchange Healthcare services to geographically remote patients	Bluetooth data transmission is slow and risky	Security of mobile agents
[17]	Healthcare	Cloud, 5G and sensors	IIS	Standard formatting of ER data Storage and retrieval of healthcare data	The proposed agent just plays the role of data storage and retrieval	Validation was missing
[18]	Patients with mental illnesses.	Chatbots/agents	SLR	Explored the role of chatbot in Psychiatric Landscape	No recommendation or solution was proposed	SLR was based on 8 studies only
[19]	Patient monitoring	WSN	Use of multi gents, SOM and DAI	Improved sensor performance in WSN	Do not discuss the role of agents in patient monitoring	The main focus is on the network not on patient care
[20]	Patient monitoring	Robots Belief-Desires-Intentions (BDI)	Multi-agent-based HMS	Bridging the gap between patient and physician decision making	If the patient is not in the position of reasoning, the agent cannot make the decision	Validation in a real- environment is missing
[21]	Indoor patients	Agent-based teamwork	Use of multi-agents	Teamwork support	Only handle indoor patients	Teamwork varies based on the patient’s complexity
[22]	Health data packets	Gateways and base station	Priority-based model for medical packet transmission	Management of packet transmission	N/A	The main focus is on management of beyond-WBAN transmission not on providing healthcare facility to aged patients

**Table 3:** Patients’ data used for case study.

Patient	Body Temperature F0	Pulse Rate (bpm)	Respiration Rate (b/m)	Blood PressureMm Hg	Gender	Weight (kg)	Blood Sugar (mg/DL)	History
Alice	101	120	7	100/70	M	80	100	Asthma
Bob	100	120	8	250/120	M	67	120	Hypertension
Jack	101	100	17	120/80	M	75	120	Normal
Mary	101	140	17	120/80	F	89	320	Diabetes

## Data Availability

Authors will be furnished on request.
